# Reporter Proteins in Whole-Cell Optical Bioreporter Detection Systems, Biosensor Integrations, and Biosensing Applications

**DOI:** 10.3390/s91109147

**Published:** 2009-11-17

**Authors:** Dan M. Close, Steven Ripp, Gary S. Sayler

**Affiliations:** The University of Tennessee, The Center for Environmental Biotechnology, 676 Dabney Hall, Knoxville, Tennessee, 37996, USA; E-Mails: dclose@utk.edu (D.C.); saripp@utk.edu (S.R.)

**Keywords:** aequorin, bacterial luciferase (Lux), bioreporter, biosensor, firefly luciferase (Luc), green fluorescent protein (GFP)

## Abstract

Whole-cell, genetically modified bioreporters are designed to emit detectable signals in response to a target analyte or related group of analytes. When integrated with a transducer capable of measuring those signals, a biosensor results that acts as a self-contained analytical system useful in basic and applied environmental, medical, pharmacological, and agricultural sciences. Historically, these devices have focused on signaling proteins such as green fluorescent protein, aequorin, firefly luciferase, and/or bacterial luciferase. The biochemistry and genetic development of these sensor systems as well as the advantages, challenges, and common applications of each one will be discussed.

## 1. Introduction

A biosensor consists of a biological recognition element that outputs a signal to an interfaced transducer capable of monitoring and measuring that signal. Biorecognition elements typically take the form of an enzyme, antibody, nucleic acid fragment, organelle, or a living prokaryotic or eukaryotic bioreporter cell, while the transducer classically exploits electrochemical, optical, piezoelectric, magnetic, or thermal measurement interfaces. The biorecognition element in its native form, or a genetically or biochemically manipulated version of it, is employed for tailored sensing of target analytes. Subsequent integration with the transducer yields a miniaturized sensing platform capable of self-contained ‘lab-on-a-chip’ detection and monitoring. Although such monitoring can be more precisely accomplished using analytical instruments such as mass spectrometry, their associated costs and complexity are often far too prohibitive for routine analyses and their size and power requirements tend to limit usage solely to the laboratory. Biosensors, with their small size, relative simplicity, rapidity of operation, and continuous, real-time to near real-time monitoring capabilities, possess unique characteristics conducive to the high-throughput and field-based or remote monitoring needs relevant to agricultural, environmental, pharmacological, and clinical sensing. Although the most popular biosensors incorporate enzymes or antibodies as their biorecognition elements, in this review we will focus on whole-cell biosensors because they do possess some interesting advantages, primary of which is the ability to indicate bioavailability—the effect and interactions the analyte has on a living system. As opposed to analytical instruments that measure only the total concentration of a target analyte in a sample, whole-cell biosensors that measure bioavailability indicate that the analyte can be assimilated by or directly effects a living organism, thereby exposing possible toxic interactions higher up the evolutionary scale (*i.e.*, humans).

## 2. Bioreporter Immobilization Methods

Perhaps the greatest difficulty in the development of whole-cell biosensors is the intimate adherence of the bioreporter to the transducer. Since the bioreporter is obligated to remain alive to perform its sensing duties, whatever mechanism is chosen to encapsulate, immobilize, or adhere the reporter cells must preserve and sustain viability. The most straightforward methods simply encapsulate bioreporters within polymers or gels such as agar, agarose, alginate, polyacrylamide, chitosan, polyvinylalcohol, and many others [1]. Their main detriments are that diffusion of the analyte through the polymer/gel often slows reaction times and that the analyte may irreversibly absorb within the polymer/gel making the biosensor a single use device. Premkumar *et al.* [2], rather than encapsulating the cells, embedded antibodies in a glutaraldehyde matrix and then attached *Escherichia coli* bioreporter cells to the antibodies. Thus, the *E. coli* cells, although anchored by the antibodies to a solid substrate, still remained free to interact with their target analytes. Sol-gels—silica and non-silica-based porous glass gels—are also popular encapsulation matrices, although more so for enzymes and antibodies than for whole cells due to the harsh reaction conditions during formation, resulting in poor cell survivability [3]. However, modified hydrolysis techniques and new sol-gel composites have demonstrated living bioreporter encapsulation for up to one year under refrigerated storage conditions [4]. Latex polymers have recently shown significant potential as bioencapsulants as well [5]. Bioreporter bacteria mixed with liquid latex can be ‘painted’ as thin nanoporous films on to solid substrates, allowed to dry, and then rehydrated when needed to reactivate the bioreporter cells. Since the films are thin (<10 μm), mass transfer limitations of target analyte are of less consequence. Shelf-life at room temperature extends from two months up to one year upon refrigeration. The bioreporter incorporated latex can also be used essentially as ink to robotically print precise arrays or matrices of encapsulated cells. Another unique polymer is the photosensitive polyvinyl alcohol-styrylpyridinium (PVA-SbQ) which can be mixed with bioreporter cells and then cured under ultraviolet light exposure. This allows precise photolithographic patterning of the polymer on transducer interfaces with subsequent fast curing [6]. Surface patterning of bacteria by these so-called soft lithographic techniques can be accomplished with a variety of other polymers and associated microfabrication methods and likely represent some of the most promising approaches to recently become available for integrating living cells with biosensor platforms. The reader is directed to an excellent review by Weibel *et al.* [7] for further information. Nanotechnology has also impacted cell immobilization through electrospinning, a process where droplets of a polymer solution such as polyvinylalcohol are electrostatically stretched into extraordinarily thin nanofibers [8]. Bacterial cells mixed with the polyvinylalcohol become entrapped within the exceptionally large surface area of the nanofiber matrix during the electrospinning process and have maintained viability after three months of storage at −20 °C in preliminary reports. Applying a less technical approach, Chu *et al.* [9] used ordinary cotton, polyester, rayon, and silk fibers pretreated with polyethyleneimine as a crosslinking agent to immobilize *E. coli* bioreporter cells that then remained responsive over a preliminary three day period. In a more unusual encapsulant, eggshell membrane, known for its excellent gas and water permeability, was used to physically adsorb *Pseudomonas fluorescens* cells albeit only for several hours within the test format described [10]. To avoid encapsulation altogether, reporter cells can alternately be maintained in continuous culture bioreactors for prolonged periods [11]. The bioreactors, often miniaturized down to milliliter size volumes, are then directly interfaced with the transducer to form the biosensor. There does exist a bit of complexity in such systems since pumps and channels are needed to deliver nutrients and remove wastes, but bioreactor-based biosensors for water quality monitoring, for example, have been successfully commercialized and implemented into on-line flow through devices such as the TOXcontrol sensor developed by MicroLAN (www.toxcontrol.com). Another means of bypassing encapsulation is to use electrokinetics to control particle motion, thereby moving and trapping biological cells within strictly defined areas [12]. A form of electrokinetics referred to as dielectrophoresis has been used to localize cells directly on a lab-on-a-chip transducer surface and such technology parallels well with the needs of whole-cell biosensors [13]. The natural ability of some microorganisms to form highly resilient spores can also be taken advantage of as a long-term storage solution. Date *et al.* [14] converted spore-forming *Bacillus* cells into bioreporters with shelf-lives of up to 8 months, and further showed that they could be repeatedly cycled between their active and dormant states with little ancillary effect on their sensing capabilities.

## 3. Whole-Cell Optical Bioreporters and Biosensor Integration

The function of the whole-cell bioreporter is to produce a measurable signal in response to a target analyte or related group of analytes. In whole-cell biosensors, this signal is typically either optical or electrochemical. For this review, we will focus on bioreporters that use optical signaling as their output to their companion transducer and refer the reader to Mehrvar and Abdi [15] for an excellent review on electrochemical biosensors. In biosensor applications, this primarily involves bioluminescent signals produced by bacterial luciferase (Lux), firefly luciferase (Luc), and aequorin and fluorescent signals produced by green fluorescent protein (GFP) (Table 1).

### 3.1. Bacterial Luciferase (Lux)

Bioluminescent bacteria are the most abundant and widely distributed of the light emitting organisms on Earth and can be found in both aquatic (freshwater and marine) and terrestrial environments. Despite the diverse nature of bacterial bioluminescence, the majority of these organisms are classified into three genera: *Vibrio, Photobacterium*, and *Photorhabdus* (*Xenorhabdus*). Of these, only those from *Photorhabdus* have been discovered in terrestrial habitats [36]. These bacteria often exist as symbiotes of other organisms, although some can be free-living in aquatic environments as well.

**Figure 1.**
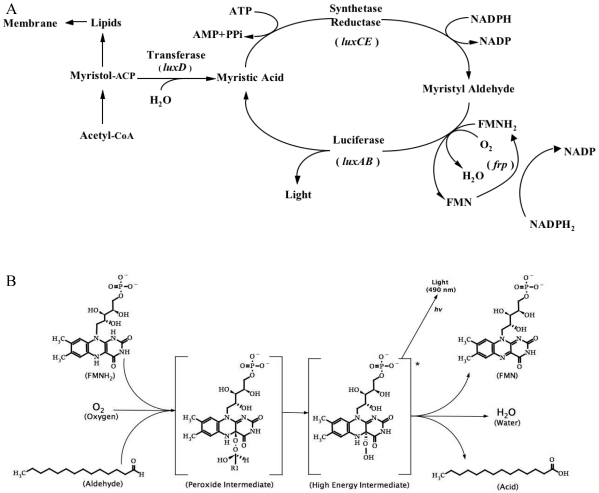
Bioluminescent reaction catalyzed by the bacterial luciferase gene cassette. A) The luciferase is formed from a heterodimer of the *luxA* and *luxB* gene products. The aliphatic aldehyde is supplied and regenerated by the products of the *luxC, luxD*, and *luxE* genes. The required oxygen and reduced riboflavin phosphate substrates are scavenged from endogenous metabolic processes, however, the flavin reductase gene (*frp*) aids in reduced flavin turnover rates in some species. B) The production of light, catalyzed by the products of the *luxA* and *luxB* genes, results from the decay of a high energy intermediate (R1 = C_13_H_27_).

Today it is well known that the bacterial bioluminescence reaction is the result of two proteins, LuxA and LuxB, that work together to produce light from the oxidation of a long chain fatty aldehyde in the presence of reduced riboflavin phosphate (FMNH_2_) and oxygen, while the remaining proteins in the *lux* operon, LuxC, LuxD, and LuxE, function to regenerate the aldehyde substrate required for this reaction (Figure 1A). However, this was not always so evident. The study of bacterial bioluminescence is rooted in the lessons of general bioluminescence. The idea that oxygen was a required substrate for bioluminescent reactions come from Robert Boyle's early experiments in the mid 1600's showing that removal of oxygen caused the cessation of light from what was either luminescent bacteria or fungi [37]. In the late 1880's when it was discovered from work in beetles that bioluminescence required a luciferase and a luciferin for function, this knowledge was applied to the bacterial system as well [38].

In 1942 Doudoroff was one of the first to observe and report on the metabolism of bioluminescent bacteria and found that all were able to tolerate oxygen, aiding in the confirmation that oxygen was required for light production [39]. Although the first published report of a bioluminescent reaction occurring outside of the bacteria occurred in 1920, it could not be reproduced reliably until 1953 when McElroy *et al.* [40] were able to consistently produce light from autolysates of *Achromobacter fischeri* cultures upon addition of FMN. At this time they also reported the requirement for a luciferin compound of unknown structure. This was the first indication that FMN was required for bacterial bioluminescence. The structure of the luciferin was later confirmed as a long chain fatty aldehyde by Strehler *et al.* [41].

This completed the list of required substrates and an understanding was established that bacterial luciferase catalyzes the production of light through oxidation of a long chain fatty aldehyde in the presence of oxygen and reduced riboflavin phosphate. The genes encoding the bacterial luciferase were first cloned and expressed in *E. coli* in 1982 [42], while the full bacterial luciferase cassette was cloned and expressed the next year [24]. In the mid 1990's the first crystal structure of the bacterial luciferase heterodimer was determined [43], giving researchers their first glimpse at the proteins that had captured their imagination for hundreds of years.

When the bacterial luciferase enzyme is supplied with oxygen, FMNH_2_, and a long chain aliphatic aldehyde, it is able to produce light primarily at a wavelength of 490 nm. There is a secondary emission peak at 590 nm, however, this is only detectable using highly sensitive Raman scattering [44]. The natural aldehyde for this reaction is believed to be tetradecanal, however, the enzyme is capable of functioning with alternative aldehydes as substrates [36]. The first step in the generation of light from these substrates is the binding of FMNH_2_ by the luciferase enzyme and until recently its active site on the enzyme was not known. It has recently been confirmed that FMNH_2_ binds on the α subunit in a large valley on the C-terminal end of the β-barrel structure [45].

In order for the reaction to proceed, the luciferase must undergo a conformational change following FMNH_2_ attachment. This movement is primarily expressed in a short section of residues known as the protease labile region—a section of 29 amino acids residing on a disordered region of the α subunit joining α-helix α7a to β-strand β7a. The majority of residues in this sequence are unique to the α subunit and have long been implicated in the bioluminescent mechanism [46]. Following attachment of FMNH_2_, this region becomes more ordered and is stabilized by an intersubunit interaction between Phe272 of the α subunit and Tyr115 of the β subunit. This conformational change has been theorized to stabilize the α subunit in a conformation favorable for the luciferase reaction to occur [45].

NMR studies have suggested that FMNH_2_ binds to the enzyme in its anionic state (FMNH^-^) [47]. With the flavin bound to the enzyme, molecular oxygen then binds to the C4 atom to form an intermediate 4α-hydroperoxy-5-hydroflavin [48]. It is important to note that this critical C4 atom was determined to be in close proximity to a reactive thiol from the side chain of Cys106 on the α subunit [45], a residue that has long been hypothesized to play a role in the bioluminescent reaction, but recently has been proven to be non-reactive through mutational analysis [49].

It has been shown, however, that C4 is the central atom for the luciferase reaction and following establishment of the hydroperoxide there it is capable of interaction with the aldehyde substrate via its oxygen molecule to form a peroxyhemiacetal group. This complex then undergoes a transformation (through an unknown intermediate or series of intermediates) to an excited state generally accepted to be a luciferase-bound 4α-hydroxy-5-hydroflavin mononucleotide, which then decays to give oxidized FMN, a corresponding aliphatic acid, and light (Figure 1B) [48]. There have classically been many theories proposed to explain the exact process required for light emission [50] that continue to expand today as technology for detecting the intermediate complexes has improved. For a review of the proposed mechanism and their strengths and weaknesses, the reader is directed to Nemtseva and Kudryasheva [48].

While the bacterial luciferase protein is all that is required to generate light in the presence of its required substrates, it is often beneficial for investigators to express other genes from the operon in order to supply the luciferase with the substrates required for its autonomous function. To accomplish this, it is necessary to co-express the *luxC, luxD, and luxE* genes. The products of these genes assemble into a multi-enzyme complex and are responsible for biosynthesis of myristyl aldehyde using components already present in the cell, thus negating the requirement to supply an aldehyde substrate exogenously.

The *luxD* gene encodes for a transferase protein and is the first to act in the aldehyde biosynthesis pathway. It is responsible for the transfer of an activated fatty acyl group to water, forming a fatty acid. During the course of this reaction the enzyme itself becomes acylated. The newly formed fatty acid is next passed off to the *luxC* gene product, which activates the acid by attaching AMP from a molecule of ATP, thereby creating a fatty acyl-AMP that remains tightly bound to the enzyme. The fatty acyl-AMP is then transferred to the *luxE* gene product via transfer of the acyl group. This protein acts as a reductase and catalyzes the reduction of the fatty acyl-AMP to aldehyde using NADPH to supply the required reducing power [36]. This allows for the *in vivo* generation of the aldehyde substrate. Because the remaining FMNH_2_ and oxygen substrates are naturally supplied by the organism, the co-expression of these genes thus allows the *lux* system to operate in a fully autonomous fashion.

#### 3.1.1. Lux biosensors and applications

Bacterial luciferase is well suited to function as a reporter gene because, when expressed with the genes required for aldehyde biosynthesis, it is capable of functioning completely autonomously with no exogenous inputs. The most basic bacterial luciferase associated reporter assays are based on determining the presence or level of bioavailability of toxic compounds. Taking advantage of the autonomous nature of the *lux* operon, bioreporters can be engineered to constitutively express light under environmental conditions. Upon exposure of the bioluminescent strain to a toxic compound, it will undergo a metabolic slowdown or death, causing a decrease in the total bioluminescent signal [51]. These assays indicate that a toxic compound is present but they do not identify what the compound is. The commonly used Microtox assay operates in this regard [16]. To permit identification, other bioreporter types are engineered to specifically respond only to certain target compounds or analytes of interest. The ability of bacteria to metabolize specific compounds is taken advantage of in these sensing strategies to create fusions of target specific gene sequences with the bioluminescent *lux* genes. Thus, when exposed to a target compound, these bioreporter cells will emit bioluminescent light signals that are either dependent on the addition of a decanal substrate if only the *luxAB* genes are used or fully autonomous if the *luxCDABE* genes are used [52]. Fully autonomous *luxCDABE*-based bioreporters have the distinct advantage of reporting target analyte presence continuously and in a real-time or near real-time format. Historically, one problem associated with real-time monitoring has been the slow turnover time of the bioluminescent reaction. Coupled with the long life of the luciferase heterodimer, this has made it difficult to resolve reporter function over short periods of time. In order to compensate for this, it has been demonstrated that inclusion of a protease tag can shorten the lifespan of the luciferase proteins and increase the temporal resolution of *lux*-based reporters [53]. Besides chemical targets, *lux*-based reporter systems have also been designed to detect biological targets, for example, food and waterborne pathogens. In these systems, a bacteriophage, or bacterial virus, is used as a carrier of the *lux* genes and its ability to infect only certain bacterial hosts is exploited as a means towards delivering bioluminescence to a target bacterium [18].

An important advantage stemming from the autonomous nature of the bacterial luminescence cassette is that, since it does not require substrate addition for expression, it can be used remotely if coupled to a proper detection device. This allows for the monitoring of compounds of interest that may be inaccessible to the researcher under normal conditions because of logistical or safety concerns [54]. Although not a detraction from the microbial applications of bacterial luciferase, it should be noted that it is the only reporter covered in this review that has historically been limited to expression only in prokaryotes. In recent years this has been challenged as the gene sequences have been altered to allow for function of the full cassette in the lower eukaryote *Saccharomyces cerevisiae* [20] and for luciferase expression in cultured mammalian cells [25].

As a truly autonomous expression system, Lux interfaces extremely well with signal transducers and has seen widespread use in biosensor applications. Fiber optic cables represent one of the easiest interfaces, with the bioreporters immobilized at one end of the cable and the other end terminating at a photomultiplier tube (PMT) or other luminometer-type device. The cable can then be inserted into liquid, solid, or gaseous samples to remotely monitor for target analytes such as heavy metals, polycyclic aromatic hydrocarbons (PAHs), or for a general assessment of sample toxicity [27,29,33,35,55]. Multi-fiber optical devices immobilized with differently target sensitive bioreporters have also been developed and field tested for multiplexed monitoring [56]. In this same vein, but perhaps more user friendly, is the Lumisens 2 instrument developed by Horry *et al.* [57] where the bioreporter bacteria are immobilized on a disposable card rather than the fiber optic cable itself. A fiber optic cable then scans each individually immobilized bioreporter to monitor for bioluminescence output in a flow-through format. Similar flow-through samplers have been constructed using bioreactors containing growing cultures of the bioreporter into which bare fiber optic cables are inserted. Upon exposure to a target analyte or toxic intermediate, the bioreporter culture yields increased (or diminished) bioluminescence that is detectable via the integrated fiber optics. Continuous, on-line water toxicity monitoring has been demonstrated using small-scale (1–2 mL) bioreactors and larger commercially available systems such as the previously mentioned TOXcontrol sensor that can be plumbed into pre-existing water lines [11]. Fiber optics have also been used to monitor bioreporter bacteria in their natural environment to non-invasively assess metabolic and physiological responses to ecosystem perturbations, for example, the addition of a contaminant [58].

**Figure 2.**
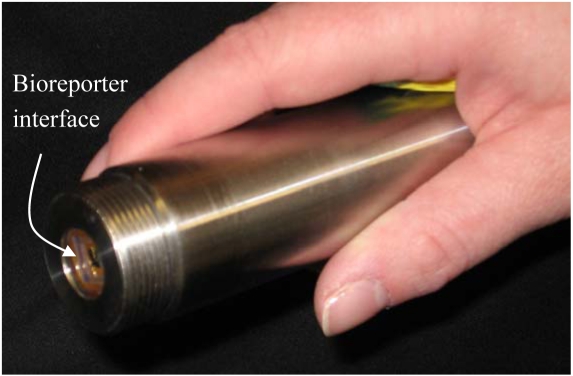
Example of a CMOS microluminometer transducer in a hand-held biosensor format. Bioreporter cells engineered to emit bioluminescent light signals are directly interfaced to the transducer element to form a compact and remotely operable biosensor.

Although functional, the requisite linkage of the fiber optic cable to a PMT or other light gathering device necessitates size and power constraints that are not conducive to miniaturization. To address this, several groups have developed different variations of chip-based microluminometers that can directly interface with the bioreporter organisms. This negates the need for a fiber optic cable to channel the signal to a transducer and instead forms an all-inclusive bioreporter-on-a-chip biosensor. This technology was first demonstrated with the bioluminescent bioreporter integrated circuit (BBIC) that consisted of a small (1.5 × 1.5 mm), low-power (3 mW) CMOS microluminometer for light gathering and a transmitter for remote data transmission [59]. Polymer encapsulants attach the bioreporters directly on to the BBIC surface or the BBIC can be interfaced with bioreporter inoculated flow-cells or bioreactors. For field monitoring, the BBIC has been incorporated into a handheld wand that operates off of an internal lithium watch battery (Figure 2) [31].

As a photodetector add on, MOEMS (Micro-Opto-Electro-Mechanical-System) can increase detection limits by minimizing system noise using an integrated heterodyne optical system (IHOS) technique that modulates bioreporter bioluminescence prior to photoconversion [60]. A MOEMS modulator/solid state photodetector interface has been tested with a Lux bioreporter and a minimum detectable signal of 10^9^ photons/sec/cm^2^ was demonstrated. To accommodate multiplexed, multi-analyte sensing on a single chip, Eltoukhy *et al.* [61] designed a 128 channel array CMOS microluminometer capable of holding and individually sensing multiple bioreporters simultaneously, thus enabling high density fingerprinting of sample chemical makeup using any of the many differently analyte-specific bioreporters available, all within a single lab-on-a-chip platform. Avalanche photodiodes (APDs) may also be of utility to bioreporter sensing as they can be designed for photon counting, much like a photomultiplier tube, but in a miniaturized standalone design [62]. APDs currently represent the most sensitive solid-state devices available and can achieve quantum efficiencies greater than 90%. However, they require higher operating voltages, generate excessive background noise that may mask low level signals generated from bioreporter cells, and their complex circuitry translates into high cost. Daniel *et al.* [22] have preliminarily tested an APD in conjunction with a stress responsive Lux bioluminescent bioreporter within a 10 μL sample chamber and demonstrated sufficient sensitivity at low part-per-million concentrations of a nalidixic acid inducer. This group has also recently developed an integrating sphere device capable of measuring absolute photon numbers emanating from bioluminescent cells, which, although too complex and fragile to serve as a biosensor, should find important utility in shaping factors fundamental to biosensor engineering such as quantum yield and minimum signal detection parameters [63].

### 3.2. Firefly Luciferase (Luc)

Firefly luciferase (Luc) is the best studied of a large number of luminescent proteins to be discovered in insects. The insects represent a large related group of bioluminescent organisms, with over 2,500 species reported to be capable of generating light [64]. While the vast majority of these luminescent reactions remain unstudied, the exception is in the order Coleoptera (beetles) where systems have been characterized for the chick beetles, railroad worms, and fireflies (predominantly *Photinus pyralis*) [65]. Fireflies produce light in an organ called a lantern, using the rapid introduction of oxygen as a trigger for luminescence in order to attract mates as well as deter potential predators [66].

The first studies of the mechanism behind insect luminescence were carried out in the late 1800's by Raphael Dubois using the ground up abdomens from the elanteridae beetle. It was Dubois who first proposed the existence of a system employing a luciferase and a luciferin for the production of light. The next advance came from Newton Harvey, who reported on the specificity of luciferase/luciferin interactions and confirmed the requirement for molecular oxygen [65]. In the mid 1900's William McElroy began what was to be a long and successful career working with firefly luciferase by discovering the requirement that ATP be involved in the luminescent reaction [67]. Based in part on these findings, his group soon proposed that the bioluminescent reaction occurred via a two step process [68] and was the first to determine the structure of the firefly luciferin as 2-(4-hydroxybenzothiazol-2-yl)-2-thiazoline acid [69]—commonly abbreviated as LH_2_ in the literature to signify reduced luciferin. In the late 1960's and 1970's the mechanism underlying the luminescent reaction was reported [70,71], as was the confirmation of the intermediate products of this proposed reaction [65]. The mechanism was finally secured in 1980 when oxyluciferin was isolated as a purified product of the LH_2_ luminescence reaction [72]. The latest advance in the understanding of firefly luciferase came in 1996 when Conti *et al.* [73] published the crystal structure of the luciferase at a resolution of 2.0 Å. This opened the door for targeted mutagenesis investigations and gave researchers the first look at the structure of this reporter protein.

**Figure 3.**
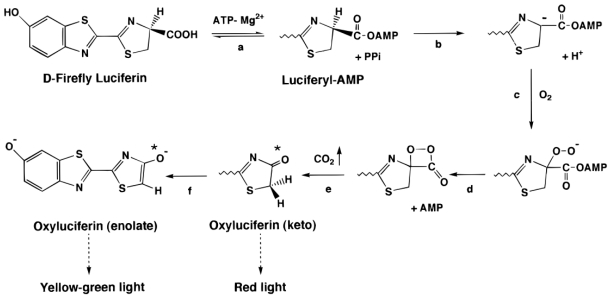
The bioluminescent reaction catalyzed by firefly luciferase. The luciferase protein holds the reduced luciferin to allow for adenylation (a). This process is followed by a deprotonation reaction that leads to the formation of a carbanion (b) and attack by oxygen (c), driving the formation of a cyclic intermediate (d). As this intermediate decays, carbon dioxide is released, forming the excited state luciferin in either the keto (e) or enolate (f) form. Used with permission from Branchini *et al.* [77].

The Luc protein catalyzes the oxidation of the reduced luciferin (LH_2_) in the presence of ATP-Mg^2+^ and oxygen to generate CO_2_, AMP, PP_i_, oxyluciferin, and yellow-green light at a wavelength of 562 nm (Figure 3). It is important to note that LH_2_ is a chiral molecule, and while both the D and L forms can bind to Luc and participate in adenylation reactions, only the D form is capable of continuing on in the reaction to generate light [65]. This reaction occurs with a quantum yield of 0.88, the highest of any characterized luminescent system with nearly one photon produced per oxidized luciferin [73]. Because of the high quantum yield, the reaction is well suited to use as a reporter with as few as 10^−19^ mol of luciferase (2.4 × 10^5^ molecules) able to produce a light signal capable of being detected [74].

It has been known since the early 1950's that the chemical reaction underlying firefly luminescence is a two-step process that first requires adenylation of LH_2_ followed by oxidation and the production of light [68]. Prior to the initiation of the reaction, the Luc protein must first bind to LH_2_. However, at this time it is not yet capable of undergoing oxidation or producing light. The first step in the generation of light is the adenylation of the bound LH_2_ with the release of pyrophosphate [75]. The function of this adenylation is to increase the acidity of the C4 proton of the thiazoline ring on LH_2_. This allows for removal of a proton from C4 causing formation of a carbanion [76]. This carbanion is then attacked by oxygen, displacing AMP and driving the formation of a cyclic peroxide with associated carbonyl group (a dioxetanone ring). As the bonds supporting this structure collapse, it becomes decarboxylated, releasing CO_2_ and forming an electronically excited state of oxyluciferin in either the enol or keto form [75].

The kinetics of this reaction can be altered by varying the concentration of the substrates, with low concentrations (in the nM range) showing steady light production and high concentrations (μM range) producing a bright flash followed by decay to 5–10% of the maximum [78]. There are multiple possible inhibitory compounds that could be responsible for the kinetic profile generated under high substrate concentrations. It has previously been shown that even though oxyluciferin is a natural product of the luciferase reaction, it is capable of remaining bound as an inhibitor to enzymatic turnover [79]. The same was found to be true of another potential byproduct, L-AMP, which can account for up to 16% of the product formed during the luminescent reaction [80]. This may, in part, explain how the addition of CoA to the luminescent reaction can result in improved performance. When CoA is added during the initial steps of the reaction, it prevents the fast signal decay normally observed, and when it is added following this decay it can promote re-initiation of the flash kinetics. This can be attributed to CoA's interaction with L-AMP to form L-CoA, resulting in turnover of the Luc enzyme and reoccurrence of the luminescent reaction [81].

Insects, and specifically beetles, that produce luminescence are quite diverse in the colors they are capable of producing. It was originally believed that the colors were the result of divergent luciferase structures, however, the sequences of four luciferase genes from *Pyrophorus plagiophthalamus* with four different emission spectra were sequenced and it was found that they shared up to 99% amino acid identity [82]. There are currently three mechanisms that have been proposed to explain the multiple bioluminescent colorations: the active site polarity hypothesis [83], the tautomerization hypothesis [84], and the geometry hypothesis [85].

The active site polarity hypothesis is based on the idea that the wavelength of light produced is related to the microenvironment surrounding the luminescent protein during the reaction. In non-polar solvents the spectrum is shifted towards blue and in polar solvents it is more red-shifted. It is questionable, however, if polarity fluctuations can account for large scale changes like those that have been observed in *P. plagiophthalamus*. The tautomerization hypothesis states that the wavelength of light produced is dependent on whether either the enol or keto form of the luciferin is formed during the course of the reaction. A recent study has reported that by altering the substrate of the reaction the keto form of the luciferin can produce either red or green light, making this hypothesis unlikely [86]. Finally, the geometry hypothesis suggests that the geometry of the excited state oxyluciferin is responsible for determining the emission wavelength. In a 90° conformation it would achieve its lowest energy state and red light would be produced, whereas in the planar conformation it would be in its highest energy state and green light would be produced. Intermediate colors would be the result of geometries between these two extremes [64].

#### 3.2.1. Luc biosensors and applications

Firefly luciferase makes an excellent reporter for the reasons previously discussed, however, the major hurdle has always been the expression of the reporter in real-time due to the requirement of a separate luciferin. Because of this, the majority of historical studies in bacteria have focused on the use of Luc outside of the cell in *in vitro* reactions, preventing the ability to detect expression in real-time. However, the bright nature and quick reaction time of Luc make it an excellent candidate for fast, large-scale applications such as immunoassays. An advantage of this type of system is that the bioluminescent signal can be correlated to the concentration of the compound of interest to allow for rapid quantification with little to no background [87].

The use of Luc *in vivo* in bacterial systems overcomes the previous detractions associated with the use of a firefly luciferase in bacterial cells, however, there is still no known way to generate the luciferin in a bacterial system *de novo*. It has recently been reported that bacterial cells can be dehydrated, which increases pore size large enough for uptake of the luciferin, and then re-hydrated while containing the luciferin without ill effects. This presents a simple and inexpensive method for introduction of the luciferin substrate. Because of the efficiency of the Luc reaction, it is even possible to visualize the location of the Luc protein spatially within individual cells using this method [26]. Another promising advance has been the isolation and characterization of the luciferase regenerating enzyme from *P. pyralis* [88]. It has been suggested that co-expression of this enzyme along with Luc could allow for continual bioluminescent production without the need for re-addition of the luciferin substrate [89].

In terms of biosensors, Luc is not routinely used since its requirement for luciferin is a hindrance to the autonomous monitoring principle of the biosensor. For example, Ikariyama *et al.* [28] immobilized *luc*-incorporated *E. coli* bioreporters in a dialysis membrane attached to the distal end of a fiber optic cable to monitor for benzene derivatives in liquid test samples. The fiber optic cable was first immersed in the sample for a fixed amount of time, and then luciferin was hand injected along with a cell lysis agent to instigate the bioluminescent response that was then subsequently measured by a PMT. Obviously, the hands-on manipulations do not contribute to an ideal biosensor format. Maehana *et al.* [30] solves the extraneous luciferin addition somewhat by incorporating microfluidics into their chip-based biosensor. Various microwells patterned onto a silicon wafer hold the sample and the bioreporter bacteria (a genotoxic sensing *E. coli* strain), and although the luciferin substrate is later added within the same microwells, one could envision separate wells and microfluidic mixing as a means towards forming an all-inclusive biosensor. Indeed, Mei *et al.* [90] describes techniques for doing so. Measurement of luminescence was also performed off-chip with a CCD camera, but again, previously mentioned microluminometers could be directly interfaced to form a true biosensor.

### 3.3. Aequorin

**Figure 4.**
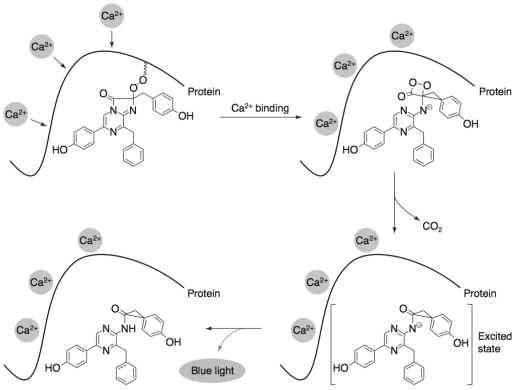
The bioluminescent reaction catalyzed by aequorin is dependent on the pre-bound coelenterazine luciferin. Upon calcium binding, the steric orientation of the luciferin is disturbed leading to a cyclization reaction that irreversibly forms a dioxetanone intermediate. As this intermediate decays, carbon dioxide is released and a singlet-excited anion is produced, followed by the generation of light at 465 nm. Used with permission from Jones *et al.* [94].

While the luminescent product of the aequorin protein has been known since man first set out to the sea, it was not until 1962 that the protein itself was first isolated [91]. Aequorin is a calcium-sensitive luminescent protein native to the jellyfish *Aequorea aequorea*. Despite knowledge of its existence there was originally much difficulty in isolating the protein because it did not use a conventional luciferase-luciferin interaction to produce light, but rather relied on the presence of calcium ions to excite a pre-bound fluorophore (Figure 4) [92]. Further study of the mechanism behind aequorin's luminescent nature was delayed until the first practical use of the isolated protein was described in 1967. It was published that aequorin could be used as a bioreporter to monitor calcium signaling in the muscle fibers of barnacles following direct microinjection [93]. This demonstration of aequorin's application lead to a renewed interest in the mechanism underlying its luminescence and set off a long history of its use as a reporter protein.

Due in part to the challenges associated with gathering large amounts of protein from the native jellyfish, it was not for another five years that the structure of the chromophore was discovered to be coelenterazine [95], the same molecule that was isolated from the luminescent squid *Watasenia* [92] and chemically synthesized by Inoue *et al.* [96] that same year. With the chemical synthesis of coelenterazine published, the major hurdle to aequorin's use was the difficulty in obtaining usable amounts of protein from the jellyfish. This was overcome several years later when the cDNA of the aequorin protein was first cloned and expressed in *E. coli* [97,98]. Following this, the crystal structure was determined in 2000 [99], opening the way to a full understanding of the structure and function of this important protein.

For successful production of light, the aequorin apoprotein must bond with coelenterazine and be triggered by calcium. The result of this reaction is the production of blue light at a wavelength of 465 nm, the evolution of CO_2_, and the conversion of mature aequorin to blue fluorescent protein (the aequorin apoprotein now bound to coelenteramide) [100]. The emission of light at this wavelength requires the generation of an excited state molecule that must be populated by a chemical reaction with at least 70 kcal/mol of exergonicity. This much energy cannot be explained simply by the binding of calcium to the protein, indicating that the luminescence reaction is proceeded by an intramolecular chemical reaction [101]. The binding of calcium functions as the trigger of this reaction, which in turn produces luminescence.

The proposed mechanism of this trigger is the result of the structural orientation of the calcium binding sites in relation to the hydrophobic residues of the protein backbone that have been shown to stabilize coelenterazine in the hydrophobic cavity. The loop structures in their associated EF-hand motifs are not properly positioned to bind calcium in their native state. Upon binding there must necessarily be changes in the spatial relationship between the coordinating amino acid residues to accommodate the calcium ion [101]. The key residues involved in this shift appear to be His169 and Tyr184, since these have previously been established as coordinating the position of coelenterazine in the hydrophobic cavity [99]. Because of the rigid stability imparted to coelenterazine by these residues, it can be expected that any rearrangement due to calcium binding would therefore result in the reorientation of coelenterazine.

The accepted chemical reaction of coelenterazine to produce light has been suggested by McCapra and Chang [102] and related to the structural changes resulting from calcium binding by Vysotski and Lee [101]. Briefly, following the structural changes imparted by calcium binding, the shared hydrogen-bonding network between coelenterazine, Tyr184, and His169 becomes disrupted. This forces His169 to become partially protonated while Tyr184 assumes a negative charge. The hydroperoxide attached to coelenterazine at C2 will then protonate Tyr184 in a reaction made possible because they share a similar pK in the hydrophobic cavity under these conditions. The resulting negative charge on the hydroperoxide then undergoes a nucleophilic attack on C3 of coelenterazine to irreversibly form a dioxetanone intermediate. This cyclization provides the energy required to drive the production of light from the overall reaction [101].

As the bonds between newly cyclized oxygens collapse, the peroxide is released as CO_2_ and a singlet-excited anion is produced. This anion is capable of emitting light directly or it may first be protonated (presumably by transfer of the proton originally transferred to His169) to produce singlet-excited coelenteramide, which is also capable of emitting light [103]. The fact that the rate of this reaction is dependent on the concentration of calcium ions has made it an attractive compound for use in reporter systems. When exposed to saturating concentrations (>100 μM), the reaction is almost instantaneous, however, when the concentration is lower, there is a relationship between the fractional rate of consumption and calcium concentration. Because only one photon is produced per reacted protein under coelenterazine limiting conditions, this rate can be used to determine the initial concentration of calcium ions [104]. Although this reaction is inhibited by Mg^2+^, it can also be triggered by Eu^2+^, Sr^2+^, and Ba^2+^, making aequorin a multifaceted reporter protein [105].

Although aequorin may be the best characterized of the calcium dependent photoproteins, it is by no means unique, nor is it the only photoprotein to be functionally employed as a reporter. More than 25 different coelenterate organisms have been shown to possess this type of protein [101], while seven have been isolated thus far: thalassicolin, aequorin, mitrocomin, clytin, obelin, mnemiopsin, and berovin [106]. Along with aequorin, mitrocomin, clytin and two homologs of obelin have published cDNA sequences [101]. All of these calcium dependent photoproteins are small, single polypeptides with molecular weights ranging from 21.4 to 27.5 kDa and all contain three calcium binding sites with affinities (k_d_) ranging from 1 to 10 μM [107]. The conserved structure and luminescent systems shared between these proteins greatly contributed to the discoveries behind their mechanism of action. Had researchers working on the luminescent systems of different proteins with similar structures and mechanisms not been able to share their discoveries, there may not yet have been an explanation for the mysterious means behind this fascinating phenomenon.

#### 3.3.1. Aequorin biosensors and applications

Historically, aequorin has found use as a calcium reporter in a variety of systems. Even recently it is still being employed in this function to monitor how various factors affect the concentration of calcium in a model bacterium [32]. However, this dynamic reporter is not limited to just calcium detection. One of the more interesting detection systems employing aequorin is the Cellular Analysis and Notification of Antigen Risks and Yields (CANARY) assay. Cultured B cells with antigens to various pathogenic bacteria are engineered to express aequorin. As a result of contact between the cell surface antigens and the bacteria of interest, an internal signaling cascade is set off that ultimately releases calcium ions and triggers the production of light via aequorin [34]. The assay has been integrated into a biosensor-like device referred to as PANTHER (Pathogen Notification for Threatening Environmental Releases). An air sampler brings pathogens in contact with the B cell reporters and corresponding signal emission is measured by a luminometer. Currently, 21 different pathogens can be detected within a three minute assay.

Aequorin is also becoming more popular as a quantitative label in binding assays. There are several attributes that make it attractive for this function, not the least of which is that it provides for sensitivity down to 10^−21^ mol while remaining free from the health hazards normally associated with the radioactive labels previously employed in this function. It has also proven itself to be quite stable over time with 85% of its original activity retained over one month of storage at 4 °C and a shelf life of greater than one year following lyophilization. In addition, the bioluminescent nature of aequorin's light production means there is virtually no background in biological samples compared to the level of naturally fluorescent molecules present in these systems [106]. Taken together, these attributes can make aequorin the perfect choice for rapid detection of low level compounds or elemental exposures in living systems.

### 3.4. Green Fluorescent Protein (GFP)

Green fluorescent protein (GFP) was first discovered during investigation into the related chemiluminescent protein aequorin from the jellyfish *Aequorea victoria* [91]. Since that time it has been realized that the *Aequorea* derived GFP is just one of a larger family of homologous fluorescent proteins capable of producing light in a variety of colors due to alterations in the covalent structure of their chromophores or differences in the surrounding non-covalent environment [108]. Despite its early discovery, the use of GFP as a research tool did not begin until after it was successfully cloned almost thirty years later [109]. However, soon after the cDNA was available, its function was validated in both prokaryotic and eukaryotic organisms by Chalfie *et al.* [110], and since that time it has been used in numerous applications including localization studies, protein expression monitoring, as a reporter gene, as a viability marker, to detect the onset of apoptosis, and many others (reviewed in [111]).

GFP has become a favored tool for molecular studies because it is autofluorescent and does not require the addition of any cofactors to properly function in exogenous systems [112]. However, it does require activation by an excitation light source before its signal can be measured. It is also resistant to heat, alkaline pH fluctuations, chaotropic salts, organic solvents, and many proteases [113], and its expression in exogenous environments is primarily non-toxic [111] with a few proven exceptions [114,115] that may be due to production of hydrogen peroxide as a by-product of synthesis [116]. However, GFP's slow posttranslational chromophore formation, oxygen requirement, and potential difficulty in distinguishing its signature from background fluorescence can be problematic [111], especially in aerobic organisms. Because of this, alternate fluorescent proteins such as those based on flavin mononucleotide are often used when developing reporters from anaerobic organisms [117]. Time, though, has proven that the benefits outweigh the challenges for most investigators, and GFP has taken its place as one of the most popular tools currently available for cellular and molecular signaling research.

**Figure 5.**
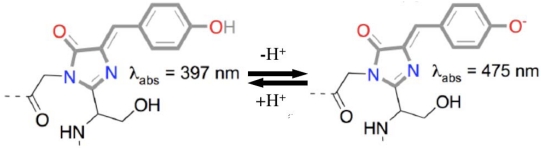
The dual absorption peaks in the GFP spectra are the result of different charge states in the GFP chromophore. The neutral state (left) is responsible for the major peak at 397 nm while the anionic form (right) is responsible for the minor peak at 475 nm. Regardless of the chromophore charge state, emission occurs at 504 nm. Adapted from Scholarpedia.org.

Wild-type GFP protein is able to absorb light at two different wavelengths. A minor peak occurs at 475 nm with the major peak at 397 nm (Figure 5). Regardless of which excitation wavelength is used, emission occurs only at 504 nm [118]. The different absorption peaks have been attributed to varying protonation states of the fluorophore, with the neutral state corresponding to the major absorption peak at 397 nm and the anionic form contributing to the minor peak at 475 nm [119]. The large shift between the major absorption peak at 397 nm and the emission at 504 nm can be attributed to an excited state proton transfer from the side chain of the Tyr66 residue of the fluorophore [120] to the carboxylate oxygen of Glu222 [111].

Based on this interconversion of the fluorophore, a three state model of photoisomerization has been put forward to explain the chemical basis for shifts in absorption. This model states that excitation of the neutral state fluorophore can cause conversion to the anionic form via an intermediate [120]. The intermediate is structurally similar to the neutral form of the fluorophore, but has become deprotonated at the phenol group of Tyr66 [111]. Excitation of the anionic form is capable of directly emitting fluorescence, while the neutral state must necessarily convert into an excited form of this intermediate prior to emission [121]. While it is possible for the neutral form to convert to the anionic form following excitation, this is not the most favorable reaction. The majority of excited, neutral fluorophores will convert briefly to the intermediate state, where fluorescence will occur, followed by reversion back to the neutral state [120]. Interconversion between the neutral and anionic states is possible, but requires both proton transfer and conformational change to occur [111]. Similarly, the majority of anionic fluorophores will revert to the ground state following fluorescent emission, but could instead undergo a conformational change to the intermediate state and then continue on to adopt a neutral charge state [120].

In a wild-type population, GFP contains a 6:1 ratio of neutral to anionic fluorophores [116], explaining why the major absorption peak is found at 397 nm. However, upon extended ultraviolet (UV) illumination this peak will begin to decrease and the minor peak will increase [122]. This behavior corresponds to the photoisomerization of the neutral fluorophore form responsible for the major absorption peak being converted into the anionic form as discussed above. While the photoisomerization characteristics of GFP can prove problematic for quantification, they do allow for the study of protein movement by excitation with intense UV light at 397 nm followed by excitation at 475 nm in order to track the movement of the photoisomerized fluorophores [123].

Following the discovery of GFP, it was quickly proven that amino acid substitutions were capable of altering its fluorescent characteristics. Since that time, versions of GFP have been developed that fold more efficiently at higher temperatures [124], avoid dimerization at high concentration [125], or fluoresce in the blue [126], cyan [127], or yellow [128] wavelengths. Homologs have since been discovered that fluoresce in the red range as well [129]. The history and development of these variants is outside the scope of this review, but an excellent classification has been made by Tsien [116] and abridged by Zimmer [111] dividing the known variants into seven classes based on spectral characteristics. When applied in concert, these variants of the GFP protein have given researchers the ability to use multiple GFP-based reporters in the same environment at the same time, improving the usefulness and range of this already dynamic protein.

#### 3.4.1. GFP biosensors and applications

Since it was first demonstrated that GFP could be expressed in *E. coli* [110], it has been used in countless experiments in organisms ranging from bacteria to cultured human cells and even commercialized for sale in designer pets. Aside from basic localization assays, the two main uses of GFP as a reporter focus on either the induction or suppression of GFP expression to indicate interaction with an analyte of interest. One of the more popular assays focusing on the suppressed expression of GFP is the determination of cell viability. Bacteria expressing GFP are exposed to compounds of interest and the severity of toxicity is determined by monitoring the decrease in fluorescent expression [17]. This allows researchers to process a large number of samples very quickly in an automated fashion. As the bacteria are killed or their metabolism is slowed by interaction with toxic substances, the overall amount of fluorescence will decrease. This type of assay has the added advantage of determining the amount of a given compound that will be bioavailable to the organism being tested.

The inverse of this type of experiment is to induce the expression of GFP as a positive result. In this case the gene encoding for GFP is placed under the control of a genetic promoter that responds specifically to the analyte of interest. This allows for visual detection when the organism is exposed to the target analyte. An advantage of this type of experimental design is that the amount of fluorescence produced can be correlated to the concentration of analyte, allowing for an approximate quantification [130]. It is also possible to use the fluorescence of GFP as a marker to isolate those members of the community showing a response by using fluorescence activated cell sorting (FACS) [131]. Using GFP to confirm interaction with a compound of interest, quantify the amount of exposure, and isolating exposed cells simultaneously illustrates its dynamic functionality in modern bioreporter research.

**Figure 6.**
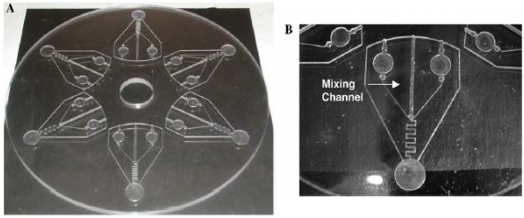
A) A lab-on-a-CD microfluidic device used in conjunction with GFP bioreporters sensitive towards arsenic. B) A close-up view of the microfluidic channeling that permits sample and bioreporter mixing. Used with permission from Rothert *et al.* [23].

Biosensor integration of GFP-based bioreporters, however, remains fairly limited due to signal output by GFP being contingent upon an excitation light source. Thus, these set-ups require one energy source to excite GFP and another to measure GFP, and the associated complexity and bulkiness are often not suitable for biosensor applications. There has been some application success using fiber optic cables where one cable is used for excitation and another for emission measurement. Shetty *et al.* [19], for example, constructed a GFP-based *E. coli* sensitive to the monosaccharide L-arabinose and entrapped it within a dialysis membrane tied to the tip of a fiber optic bundle. Fibers terminating at a tungsten lamp served as the excitation source while separate fibers terminating at a PMT served as the detector. Immersing the *E. coli* entrapped sensor end of the fiber bundle in liquid was then shown capable of detecting L-arabinose at varying concentrations. To improve sensitivity, Knight *et al.* [21] bypassed fiber optics by interfacing a PMT directly with a flow-cell containing a yeast-base GFP bioreporter sensitive to DNA damaging genotoxic compounds. An argon laser provided the excitation source. Realizing the necessity for miniaturization and less complexity, new fluorescence detection techniques based on small footprint biosensor compliant platforms are becoming somewhat available. Complementary–metal–oxide–semiconductor (CMOS) photodetectors better tuned to the green light signature provide enhanced detection [132], as do avalanche photodiodes, while tightly focused laser beams provide excitation down to the single cell level [133]. However, incorporating all necessary components into a true biosensor format remains challenging. Rothert *et al.* [23] developed a 12 cm diameter microfluidics-based lab-on-a-compact disk (CD) device that microcentrifugally moved and mixed microliter volumes of water test samples with a GFP bioreporter sensitive to arsenic (Figure 6). Although sensing was accomplished with a fiber optic probe positioned above the CD, the device could likely be easily reconfigured to accommodate a chip-based sensor to promote further miniaturization.

### 3.5. Alternative Bioreporter Systems

The reporter systems covered in detail in this review represent the majority and most popular signaling proteins commonly used by investigators for optical biosensing applications. Along with homologues to the proteins reported here, there are myriad other systems capable of either acting as a reporter or being complexed to a transducer for biosensor monitoring. These include β-galactosidase, β-glucuronidase, catechol 2,3-dioxygenase, chloramphenicol acetyltransferase, the ice nucleation protein InaZ, Uroporphyrinogen III methyltransferase [134,135] and more recently, engineered infrared-based fluorescent proteins [136]. Each of these signaling elements has its own advantages, disadvantages, and rich history of discovery and use just as the ones covered in this review. We, as investigators, are fortunate to work in a time when so many diverse reporters are available. But since centuries ago when the first work began on the study of these interesting systems, it has never stopped. New discoveries will assuredly occur to develop novel reporter systems and add to this already impressive and dynamic range of biosensing tools.

## Figures and Tables

**Table 1.** Whole-cell bioreporters referenced in the text. Reporters are grouped by the bioluminescent or fluorescent system exploited. Reporter cell type is listed above the compound(s) detected and references refer to original publications detailing construction and sensitivity of each reporter construct.

**Bacterial Luciferase (Lux)**		**Green Fluorescent Protein (GFP)**	
*V. fischeri*	[16]	*E. coli*	[17]
Various toxins		Various toxins	
Bacteriophage	[18]	*E. coli*	[19]
Pathogenic Bacteria		L-arabinose	
*Saccharomyces cerevisiae*	[20]	Yeast	[21]
Androgenic compounds		DNA damage	
*E. coli*	[22]	*E. coli*	[23]
Nalidixic Acid		Arsenic	
*E. coli*	[24]	*Bacillus* sp.	[14]
Various stressors		Arsenic/Zinc	
HEK293 mammalian cell line	[25]	**Firefly Luciferase (Luc)**	
	
Whole animal imaging		*E. coli*	[26]
		Gene expression	
*E. coli*	[27]		
Polycyclic aromatic hydrocarbons		*E. coli*	[28]
		Benzene	
*Alcaligenes eutrophus*	[29]		
Heavy metals		*E. coli*	[30]
		Various toxins	
*Pseudomonas putida*	[31]		
Microbial volatile organics		**Aequorin**	
	
		*E. coli*	[32]
*Pseudomonas fluorescens*	[33]	Calcium	
Naphthalene/salicylate			
		B Cells	[34]
*E. coli*	[35]	Pathogenic Bacteria	
DNA damaging agents			
